# The influence of the Covid-19 pandemic on municipal meeting places arranging group exercise for older persons

**DOI:** 10.1080/17482631.2023.2235130

**Published:** 2023-07-27

**Authors:** Daniella Dinse, Maria Haak, Marie Nilsson, Staffan Karlsson, Ulrika Olsson Möller

**Affiliations:** a The Research Platform for Collaboration for Health, Faculty of Health Sciences, Kristianstad University, Kristianstad, Sweden; b School of Health and Welfare, Halmstad University, Halmstad, Sweden; c Department of Health Sciences, Lund University, Lund, Sweden

**Keywords:** Aged, Covid-19, health promotion, meeting places, municipal government, person-centredness, physical activity, stakeholder participation

## Abstract

**Purpose:**

Many municipalities today, together with other stakeholders, offer group exercises for the older population via municipal meeting places, focusing on promoting good health. During the Covid-19 pandemic, these group exercises either continued in a modified form or ceased. The aim of this study was to explore involved stakeholders’ experiences of group exercises for older persons arranged via municipal meeting places during the Covid-19 pandemic.

**Methods:**

Six online focus group interviews were conducted with 25 stakeholders, such as decision-makers and representatives from the non-profit sector, from seven municipalities in Sweden. Data were analysed using thematic analysis.

**Findings:**

The collaboration around the group exercises was challenged due to affected communication and decision-making. The stakeholders described the importance of adapting and finding new ways to offer group exercise. Furthermore, the re-arranging of group exercises created concerns about the well-being of the older persons but also happiness with the older persons ability to act for their own well-being during the pandemic.

**Conclusions:**

This study highlights the importance of the municipalities exchanging experiences, making the older persons more involved in the decision-making process, enabling a person-centred encounter with the older persons when exercising in groups, and strengthening supportive environments by sharing the ownership of arranging the group exercises with the older persons.

## Introduction

Despite the well-known health benefits of physical activity for persons aged 65 years and older (de Labra et al., 2015; Marcucci et al., 2019; Taylor et al., 2004), not all of them reach the recommended level to achieve health benefits (World Health Organization [WHO], 2018). According to the WHO (2022) at least 2.5 h of moderate to vigorous physical activity per week is recommended. Therefore, it is important to create supportive measures and environments enabling older persons to be physically active according to their conditions (Langhammer et al., 2018; WHO, 1986). Following the recommendations of Swedish authorities, many municipalities today, in collaboration with other stakeholders, offer physical activity through group exercise for older persons over the age of 65 via municipal meeting places, with a focus on promoting healthy ageing, including for example social fellowship, a meaningful everyday life and good physical and mental well-being and health (Folkhälsomyndigheten, 2022). However, these activities were affected by the Covid-19 pandemic.

Physical activity is an overarching concept, which is defined as any bodily movement carried out by the muscles that requires energy (WHO, 2022), and has the subordinate concept of exercise, which means planned, structured, repetitive, and purposeful physical activity with the goal of improving or maintaining physical fitness (Caspersen et al., 1985; Dasso, 2019). With an ageing population worldwide, health-promotive and preventive interventions, such as physical activity, are of great importance for healthy ageing (WHO, 2018). Physical activity is included in the concept of health promotion, which is the process of enabling both the population and the individual to increase control over and improve their health, and the concept of disease prevention, which involves the measures taken to prevent the onset of disease or limit its development (WHO, 1986; WHO, 2021). Physical activity for older persons can improve the quality of life (Halaweh et al., 2015) and contribute to reduced need for health care (Public Health Agency of Sweden, 2022; Woolcott et al., 2010). Therefore, it is of the utmost importance to explore what happens if arranged physical activities for older persons change or cease.

In Sweden, the municipalities are responsible for promoting an active and meaningful life for older persons in fellowship with others (Socialtjänstlagen [Social Services Act] SFS 2001:453 2001). To be able to create health-promoting living conditions and environments, the municipalities are responsible for collaborating with other stakeholders, such as other community bodies and the non-profit sector. Such collaboration takes place via municipal meeting places which focus on health-promoting interventions to create more opportunities for older persons to improve their health by getting support in making changes to their lifestyle habits. In such health-promoting work the various stakeholders such as the municipality, the non-profit sector and other organizations arranging group exercises via the meeting places are of great importance. Due to them, the municipalities have a greater variety and range of group exercises today to offer to older persons (Folkhälsomyndigheten, 2022).

The Covid-19 pandemic that started in March 2020 impacted life worldwide and led to changed prerequisites at both individual and societal level. The management of the Covid-19 pandemic varied between countries, for example some countries instituted full or partial lockdowns (Han et al., 2020). Sweden had no lockdown; instead, the authorities decided on general restrictions that applied to the entire population to slow the spread of the Covid-19 disease. The main restriction was social distancing (Coronakommissionen, 2020), meaning not to be socially distant from others but to keep a physical distance from anyone outside one’s household (WHO, 2020; WHO, 2022). Persons over the age of 70 were also urged to limit their social contact and to avoid being in places where people gathered (Folkhälsomyndigheten, 2020a). These special restrictions for persons over the age of 70 continued for about seven months (Coronakommisionen, 2020). The restrictions followed the development of the pandemic and therefore changed continuously during it and, in consequence, impacted various health-promoting activities within the community targeting older persons. The stakeholders, such as group exercise coordinators, group exercise leaders and representatives from the non-profit sector involved via the meeting places could no longer arrange activities for older persons as before. This in turn led to changed opportunities for the older persons to participate. The restrictions of social distancing and the special restrictions for persons over the age of 70 during the pandemic caused planned group exercises to rapidly decrease. Thus, from the stakeholders’ perspectives there is a need to explore how measures taken due to the pandemic influenced group exercises arranged via the meeting places.

A decrease in physical activity has been shown to have a negative impact on older persons, who are usually more inactive compared to younger persons and more prone to fragility and muscle loss (Roschel et al., 2020). Previous research before the pandemic (Cunningham et al., 2020) showed that inactive older persons had, an increased risk of mortality, fractures, recurrent falls and functional limitation, and experienced reduced quality of life and reduced cognitive ability (Cunningham et al., 2020). A later study showed that older persons who were physically active during the pandemic reported better physical health, while the older persons with increased sedentary time reported lower physical and mental health and vitality (Cheval et al., 2021). Reduced levels of physical activity during the pandemic for older persons are likely to have further negative physical and mental health consequences (Brooke & Jackson, 2020; Brooks et al., 2020).

Accordingly, a new situation arose during the pandemic when many health promotive interventions, such as group exercise for older persons, changed or ceased. Exploring such a change could lead to a greater understanding of how the prerequisites for arranging the activities changed regarding the content and the need for these health-promoting interventions, as well as how to better design supportive interventions and environments so that older persons are less affected if the group exercise activities cease for various reasons. As the stakeholders have knowledge of arranging and designing the group exercises via the municipal meeting places it was important to explore their perspectives. Therefore, the aim was to explore involved stakeholders’ experiences of group exercises for older persons arranged via municipal meeting places during the Covid-19 pandemic.

## Methods

### Design

This study has a qualitative design using an inductive approach, and a focus group methodology according to Krueger and Casey (Krueger & Casey, 2015) was applied.

### Context

This study includes seven municipalities in southern Sweden. Within the municipalities there are units engaged in preventive work to strengthen the health of the municipal residents. The meeting places are part of the preventive unit, and the basic concept of the meeting places is to offer activities together with the non-profit sector to improve the health of community-dwelling older persons. The meeting places are in designated premises and offer different forms of activities such as social, cultural, and physical activities. However, this study only explores physical activity, i.e., group exercise, which is arranged via these meeting places.

The basic concept for the meeting places is similar between the municipalities, but the structure may differ. The majority of the municipalities have one or more meeting places in the same facility as a nursing home while others have their own facilities for this purpose. The meeting places are geographically dispersed within a municipality. Two municipalities have several employees, i.e., municipal stakeholders, involved in the meeting places, while the others have only a few or none. Three municipalities mostly offer activities by themselves, while the other municipalities also collaborate with other stakeholders, for example, private stakeholders, adult education associations, sports associations, pensioners’ associations, and individual volunteers. In one municipality, group exercises are only arranged through non-profit associations.

There is variety in the types of group exercises offered. All municipalities participating in this study offer walking/hiking and exercise gymnastics in groups before the pandemic. Other common group activities are dance, balance exercises, Qigong, sports schools for older persons, strength exercises and water aerobics. The target group for these interventions are persons over 65 years, mainly community-dwelling older persons.

At the beginning of the pandemic in the spring of 2020, the meeting places were totally or partly closed. A majority of the municipalities in this study only closed the meeting places for a few weeks and could then open to some extent again. At the longest, one municipality offered no activities via the meeting places for about 16 months. The meeting places located in the same building as a nursing home were usually closed for a longer time due to the requirements of the pandemic restrictions.

### Sample

A total of nine geographically nearby municipalities were contacted, and seven arranged group exercises for older persons via the meeting places. Each of them has one or more representatives in this study. Five of the municipalities were rural and two were urban. A total of 25 stakeholders participated in the focus group interviews (Table I). One group consisted of managers and managerial support from the municipalities’ preventive units. The managerial support provided assistance that was a basis for decisions, and the managers made decisions and led the organization. The managers who participated were first-line managers of the preventive units and heads of department to which the preventive units belonged. A second group was municipal employees, such as licenced practical nurses and elderly educators, who had the role of coordinating activities. A third group consisted of group exercise leaders, such as dance instructors and licenced practical nurses, with experience in leading group exercises. A fourth group consisted of stakeholders who were employed by adult education associations, sports associations, and private stakeholders employed at health clinics. A fifth group consisted of persons from the non-profit sector such as members of various pensioners’ associations and individual volunteers who arranged group exercises via the meeting places. In some cases, some stakeholders could have two different roles, for example coordinating and leading the group exercise and then the stakeholders themselves decided which focus group to attend.

**Table I.** Characteristics of focus group participants.

Characteristics	*n* = 25
Age	
Mean (min-max)	54.4 (34-92)
Gender	
Women (%)	19 (76)
Men (%)	6 (24)
Stakeholders	
Group 1 a	
Decision-makers	2
Managerial support	1
Group 1 b	
Decision-makers	3
Managerial support	0
Group 2	
Coordinators	5
Group 3	
Group exercise leaders	5
Group 4	
Private stakeholders	1
Sport associations	2
Adult education associations	1
Group 5	
Pensioners’ associations	3
Individual volunteers	2
Experience in a stakeholder	
role in years, md (min-max)	
Group 1	5,7 (3–15)
Group 2	6,5 (1-13,5)
Group 3	6 (2.5–15)
Group 4	3,5 (2–11)
Group 5	6 (1–10)

Inclusion criteria were stakeholders who in some way arranged different group exercises via municipal meeting places before and/or during the pandemic. To recruit stakeholders a combination of purposive sampling and snowball sampling was used. When one person in a municipality had been contacted, this person was asked who else was connected to the group exercises via the meeting places. This continued until no more persons were mentioned.

### Data collection

The stakeholders were placed in homogeneous focus groups since a common frame of reference and shared experiences can facilitate the discussions during an interview (Ivanoff & Hultberg, 2006). Furthermore, having different perspectives between groups also provides variety in the sample, which is important in the focus group methodology (Krueger & Casey, 2015).

Due to the pandemic the focus group interviews were conducted and recorded via an online video communication platform. All stakeholders were invited to test the online video communication platform one week before the focus group interviews, and four did. All stakeholders were given information about the importance of sitting in a calm environment without distractions from other people. If this was not possible, they were asked to use a headset. All stakeholders chose to have the video camera on for a good interaction.

The plan was to provide five focus groups, but since there was a dropout and only a few stakeholders in the focus group with decision-makers and managerial support (group 1 a, *n* = 3), it was decided to have another focus group with decision makers and managerial support (group 1 b, *n* = 3). This was done to ensure variety in the material. In total, six focus groups were conducted between June and November 2021.

The focus groups were conducted with one moderator and one assistant moderator according to Krueger & Casey (2015). The focus group interviews started with the moderator having a general conversation and giving information about the study, the aim and how to use the online video communication system. First, there were a general request: Please tell us about the group exercise activities that you are involved in via the municipal meeting places. An interview guide consisting of the themes “challenges during the pandemic” as well as “opportunities and lessons learned during the pandemic” was used (see Supplementary file 1). The moderator guided the discussions to ensure that appropriate themes were discussed, and all stakeholders were encouraged to share their perspectives. Prompting questions were used, both by the moderator and assistant moderator, to deepen the discussion and to obtain additional information. As recommended by Krueger and Casey (2015), the assistant moderator took field notes during the interviews. Themes, key points, and the interaction in the group were noted. The recorded interviews lasted between 83 and 104 minutes but consisted also of other questions not reported in this study. After the focus groups, the moderator, and the assistant moderator reflected on their experiences of each session and wrote reflective notes.

The first and last authors led four of the focus groups as moderator or assistant moderator. The second and third authors participated in two of the interviews as the first and last authors were known to the stakeholders.

The first interview was transcribed verbatim by the first author, and the other five by a transcriber. All were then validated by the first author by comparing the recorded interviews with the transcribed text. The data material was handled confidentially, and the stakeholders were de-identified in the study.

### Data analysis

**Figure 1.**
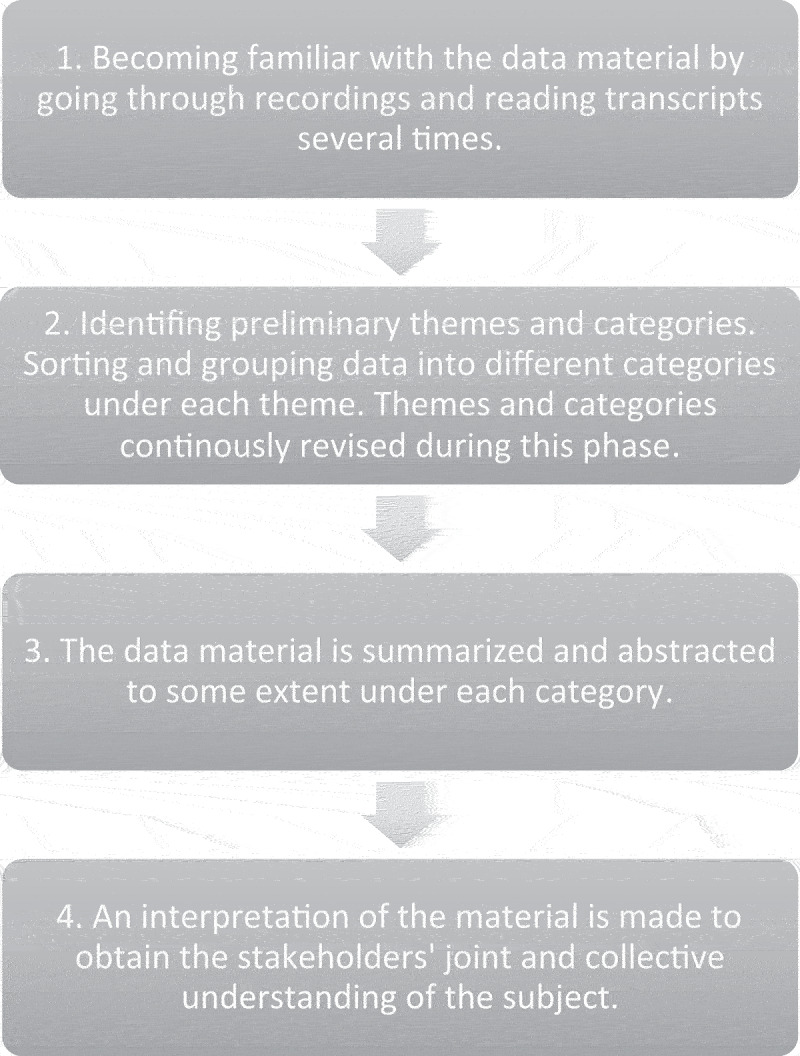
Analysis process.

The analysis was based on a method described by Krueger (Dahlin Ivanoff & Holmgren, 2017; Krueger, 1988). Through the discussions, the group perspectives were shaped and reshaped and formed the basis for a collective perception of the research topic. The notes taken during and after each focus group supported the interpretation process. The data analysis was performed according to the following steps (Figure 1). First, to gain an understanding of the data material, the recordings were gone through, and the transcript was read several times. Second, based on the aim of the study, the first and second authors separately identified preliminary categories and themes. The researchers then compared the findings with each other and sorted and grouped the material into different categories and themes in NVivo 2022 software. Themes and categories were continuously revised to ensure they matched the meaning of the data material. Third, the data material was summarized and abstracted under each category. This was then the basis for the fourth step, which was the interpretation of the material (Dahlin Ivanoff & Holmgren, 2017; Krueger, 1988), where the purpose was to understand the meaning of what had emerged during the stakeholders’ discussion to gain their joint and collective understanding of the topic. In this fourth step, the first, second, third and last authors participated.

To ensure that the findings matched the stakeholders’ understanding of the research topic, a member check (Lincoln & Guba, 1985) was carried out where the preliminary findings were presented at two oral presentations on site at Kristianstad University. All stakeholders were invited, and in total, 11 participated. One or more representatives presented each focus group. The stakeholders reflected on and agreed with the findings. For the researchers, the understanding of the findings was verified and deepened after the member check.

### Ethical considerations

The study was approved by the local Health Sciences Ethics Council at Kristianstad University (Dnr: U2021-2.1.12-1060). The study was conducted in accordance with the Declaration of Helsinki (World Medical Association, 2013). Written and oral informed consents were obtained from all stakeholders before the focus group interviews.

## Findings

**Figure 2.**
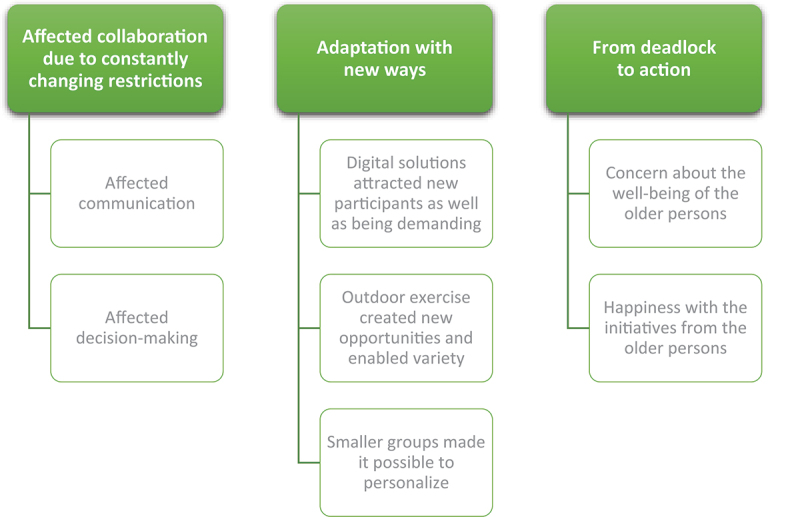
Themes and categories.

The stakeholders said that the prerequisites for arranging group exercises via the meeting places changed due to the restrictions connected to the pandemic. At first, the meeting places had to be closed completely and the stakeholders described it as a deadlock. The collaboration between the stakeholders was also affected. This is presented in the first theme *“Affected collaboration due to the constantly changing restrictions”*. Despite these challenges, the stakeholders described the importance of taking advantage of the opportunities by adapting and finding new ways to offer group exercises via the meeting places. This is presented in the second theme: *“Adaptation with new solutions”*. Furthermore, it was not only the stakeholders who were affected by the changed prerequisites or who acted to find new ways. The third theme *“From deadlock to action”* presents the stakeholders’ experiences of how the older persons’ well-being was affected as well as their ability to act during this time (Figure 2).

### Affected collaboration due to constantly changing restrictions

The constantly changing restrictions affected the collaboration between the stakeholders. Maintaining already established collaboration during the pandemic was perceived as challenging. Before the pandemic, the established collaboration, including well-functional communication and well-functional decision-making, had been of great importance for stability and variety in terms of the group exercises. Such collaboration was therefore challenged by the restrictions during the pandemic in terms of affected communication and affected decision-making.

#### Affected communication

The communication regarding the meeting places was described as challenged and affected in three different areas, all of which in turn affected the collaboration.

Communication was affected by the number of continuously updated guidelines that needed to be communicated quickly. The amount of information on various guidelines was described as large, rapidly changing, and coming from different authorities. Communicating information to such an extent from so many authorities to so many different involved parties had not been experienced before and was described as challenging. There was much information to take in, but also much information that quickly needed to be passed on to involved stakeholders and to the participating older persons via the meeting places.

It was challenging to maintain contact with all stakeholders who arranged group exercises via the meeting places. The previous regular contacts and the exchange of information that the municipal stakeholders had with the other non-municipal stakeholders decreased or ceased during the pandemic, for example when some of the stakeholders received other assignments. If this happened to the person responsible for the contact with involved stakeholders, the contact ceased completely. The lack of communication between the stakeholders was challenging. On the one hand, it led to the municipal stakeholders losing stakeholders who could offer group exercises. The non-municipal stakeholders, on the other hand, found themselves uncertain about what currently applied regarding arranging group exercise and also about the future plans for the group exercises. As a consequence, the non-municipal stakeholders said that they could not influence the planning and decisions regarding the group exercises. Furthermore, the stakeholders emphasized the need to improve communication between all stakeholders to ensure effective communication in a similar future situation.

Interview person [Ip] 11: (…) Above all, what I am experiencing right now is that the municipality made the decision to close down the meeting places early on when Corona came. But I haven’t heard a peep from the municipality since then. And I still don’t know what applies going forward. I feel a little bit that things have fallen a bit now, during the pandemic. But we’ve also changed a bit who sits on posts and so on, so it may have fallen a bit between chairs for a lot of different reasons, but… Yes, so it’s constantly a job where you have to keep those relationships going with specific [stakeholders], I feel, in order to be able to… like, to ensure a good collaboration and not fall between different chairs and so on. It depends in some way very much on the individual contact, we feel, or I feel, that it’s so for us as a [stakeholder who collaborates with the municipality].

Ip 9: I can probably agree that this thing about the individual contact is important; the contact person we had was there on a temporary basis and then when she quit… then there was also silence from the municipality’s side. And then the pandemic came and… but now they have contacted us again for a slightly bigger project, but that’s probably what [Ip 11] says there, that it’s largely the individual in the municipality who influences whether it ends or not, whether it’s driven or falls between chairs.

(Focus group no. 4)

Maintaining contact with the older persons was challenging. Before the pandemic, contact was mostly maintained when the stakeholders met with the older participating persons via the meeting places. However due to the restrictions during the pandemic, other ways of communicating had to be found, for example, through social media and video conference systems. This was challenging because it was perceived to be a one-way communication and it was uncertain whether important information reached the older persons or not. The stakeholders also said that the general assumption was that the pandemic would be brief, which led to the older participating persons being contacted only at the beginning of the pandemic but not later on. This was described as a shortcoming and the stakeholders realized in retrospect that more continuous contact with the older participating persons would have been important as it would have made the older persons more involved in the process of reopening the meeting places during the pandemic.

#### Affected decision-making

Three different areas about decision-making regarding the group exercises at the meeting places were described as affected by the new situation that the pandemic and its restrictions entailed.

The decisions needed to be balanced to take account of both the wishes of the older persons and the current restrictions. At first, it was considered necessary to close the meeting places to avoid accelerating the spread of Covid-19. As time went on, the understanding changed and instead it became important to make decisions about opening the meeting places again to counteract the negative impact on the well-being of the older persons. This understanding came from the fact that the older persons continuously conveyed to the municipal stakeholders that they had become inactive, felt lonely and they therefore wanted to start group exercise via the meeting places again as soon as possible. Their wishes were described as important to be responsive to. Furthermore, when some group exercises were reopened, many wanted to participate despite the restrictions, which indicated a good response from the older participating persons. Such a response led to decisions being made to increase such group exercises that met the wishes of the older participating persons but also were in line with current restrictions. However, it was experienced as challenging to constantly balance between making decisions based both on the authorities’ guidelines and the older persons’ wishes.

Decision-making was influenced by the fact that the guidelines left room for interpretation, which could lead to uncertainty and disagreement during the decision-making process. Consequently, the municipalities interpreted the restrictions differently, where some municipalities had ongoing group exercises while other municipalities did not. The stakeholders said that fear of spreading the Covid-19 disease was in some cases so great that they did not dare to arrange group exercises even if it was possible to some extent. Differences were also described in decisions regarding various activities within a municipality where, for example, the older persons could exercise at private gyms while at the same time the meeting places were closed for exercise. Thus, as the decisions differed between municipalities and between other activities within the same municipality, it led to uncertainty about whether the right decision had really been made. Despite this uncertainty the lesson the stakeholders learned for the future in the event of a similar situation was to dare to take more local and adapted decisions according to the circumstances of each individual municipality. A prerequisite for this decision-making was having clear and concrete guidelines from the authorities.

The perception was that decision-making was slowed down by a wait-and-see attitude among many, which was based on a belief that the pandemic would soon be over. The pandemic was a completely new situation, and no one had experience of what the outcome of a decision would be. Such an uncertain and slow decision-making process was described as affecting the decisions about meeting places’ group exercises as well as collaboration between involved stakeholders. The stakeholders therefore said that in a similar event in the future, it would be important to make faster decisions.

Ip24: (…) I think that many people believed that the pandemic would disappear quickly, and that “by summer there will be no Covid”, even after [the state epidemiologist] said that “we’ll have to live with this for a while”. This collective delusion persisted, with people saying “when the summer comes, Covid will disappear” or “but until the fall” or “when everyone has received the first dose of vaccine” and so on. Especially in 2020, we waited a long time to make decisions because we didn’t understand the seriousness and scope of the situation. Now, we know that we need to make quick decisions.

Ip25: And I also think we have a better idea of what decisions we need to make.

Ip24: Mm.

Ip25: It was such a thing, I thought like many decisions that this whole situation fell naturally back on me as a manager and Sweden is a country where we… if you look at the decisions made by the government and the Public Health Agency, they gave a lot of room for interpretation, to be honest, so you could sort of… and that also means that we had to make decisions at the local level based on our instincts, taking a chance and hoping that the decisions were correct. And next time, we’ll have experience, which means that the decisions will be much more accurate and will happen much, much faster.

Ip24: Mm.

Ip25: And we can also take like … dare to also take more local decisions faster next time, I think.

(Focus group no. 1)

### Adaptation with new solutions

The stakeholders described the importance of adapting and finding new solutions so they could arrange group exercise via the meeting places despite the restrictions. Some methods were already used before the pandemic, such as digital solutions and outdoor exercise, but on a small scale. These ways of offering group exercise could easily be adapted to current restrictions and therefore took on a greater role during the pandemic and were considered to facilitate exercise for the older persons. Furthermore, before the pandemic, it was common to arrange groups with many participants via the meeting places, but the restrictions implied that only a limited number of participating older persons could meet to exercise together. This led to the need for a new solution, which was to arrange group exercise in smaller groups via the meeting places. The adapted and new solutions described by the stakeholders were digital solutions, outdoor activities and smaller groups.

#### Digital solutions attracted new participants as well as being demanding

Using digital platforms to arrange group exercise via meeting places during the pandemic was described as a good opportunity given the current restrictions. Various exercise programmes were established and implemented digitally, either live or pre-recorded. The stakeholders said that they mainly reached the older persons who were comfortable with the digital technology. Although this generally meant a smaller number of older participating persons, it was still better than having no persons at all. Additionally, new participating older persons who previously had never exercised via the meeting places were attracted to the digital exercises. It was also described that the older participating persons became more digitally aware and developed their digital technology knowledge more as time went on during the pandemic. Such digital awareness facilitated and enabled exercise for the older persons who used the digital platform. When the older persons exercised live via the digital platform, social interaction and fellowship were also made possible, which was otherwise difficult during the pandemic. The stakeholders said that the older participating persons expressed appreciation for the digital platforms and wished that this would continue even after the pandemic.

Ip 3: And we have also said that about our digital activities, because we have learned that they really want to keep these digital activities even if we open up the meeting places, or when we do… that we should keep them digital because we have encountered new participants which we otherwise might not have done, those who don’t come to the physical meeting place but come to the digital one instead.

Moderator [M]: It’ll be a complement.

Ip 3: Yes… [gets interrupted]

Ip 2: We have encountered that too… got many new participants.

Ip 3: Yes. [Ip 1 and Ip 4 nod in agreement]

(Focus group no. 3)

The digital platforms were seen by the stakeholders as a complement to meeting physically, as before the pandemic. However, the digital platforms could be experienced as time-consuming, especially for those stakeholders who needed to learn the digital technology themselves. It was also perceived as easier to arrange digital activities of a social nature compared to exercise, and therefore, it was important to uphold and continue to develop the digital platforms to a level that suited the older persons who wished to exercise via the digital platforms.

#### Outdoor exercise created new opportunities and enabled variety

Arranging exercise outdoors was a form of adaptation and was considered beneficial for the older persons. Several group exercises that were previously held indoors could immediately be moved outside and the responses from the older persons were very positive. Newly participating older persons came and they came despite sometimes having to travel a long way, or despite cold and bad weather. The stakeholders perceived this to show that the older participating persons really appreciated being out in nature and that they really wanted to continue to exercise via the meeting places despite the pandemic. Although some older persons with balance difficulties were excluded from the outdoor exercise due to the more unstable ground outdoors, the attendance at the outdoor exercise was good. The positive response from the older participating persons led to an expansion of outdoor exercising during the pandemic.

Arranging group exercise outdoors was perceived to be favourable because the outdoor context enabled a variety of exercises to be performed and provided more opportunities to do so as they were not limited to specific premises. Before the pandemic, the stakeholders said that generally more social activities were arranged via the meeting places, but they perceived that, both during and after the pandemic, there was a need to arrange further group exercises, preferably outdoors as these were considered particularly beneficial. Therefore, opportunities were now seen when planning ahead for the meeting places, namely to make a fresh start by removing less well-functioning activities and instead introducing new activities, such as outdoor exercise. The common experience was that outdoor exercise would continue even after the pandemic and on a larger scale.

Ip 8: (…) But then I would like to say one thing that we have now learned during the pandemic, and it’s that activities don’t need to be held indoors. We have learned a lot from this; that these four walls should not limit us, we must be able to think bigger than that.

Ip 6: Mm.

Ip 8: And that we can stay both inside and outside the premises and feel these four walls don’t limit us. There, I think we have had such a… or I have had such a feeling in [my own municipality], that the meeting places are… there are a number of meeting places and so it’s limited to these four walls and we have somehow not succeeded … we have succeeded to some extent, but not to the extent that I would like, to break down those walls, but we have succeeded now during the pandemic, so now… so both… both the employees [at the meeting places] and the participants have realized now during the pandemic that yes, you don’t need premises to carry out these activities, because it’s so easy… and that’s what’s mine… a little bit my fear now when we open up again and the restrictions disappear, that we crawl into these small warm hovels and sit down again and I’m convinced that the employees [at the meeting places] feel it too. They don’t want this, but they want to continue with this openness and then it will be open [meeting places] and I believe in that a lot. And then if you think specifically about physical activity, the outdoor part is an incredibly beneficial part [the sound disappears to some extent] (…) much better than inside the premises.

M: Sorry, we didn’t really hear the last thing you said.

Ip 8: No, I mean, the outdoor environment is incredibly favourable for activities, especially physical activities.

Ip 6: Mm. I agree. We had a discussion, some said “yes, but it’s raining, or the wind is too cold” and… but I said, “they [the participants at the meeting places] are after all out and about in all kinds of weather and have done so throughout the pandemic year”, what is that … they are no worse than we are, they have learned this. And then I mean you can’t lock people up and say because you’re old you should sit indoors or you should have gymnastics inside, you can do them outside.

Ip 7: Yes, I can only agree.

(Focus group no. 1)

#### Smaller groups made it possible to personalise

In line with current restrictions, smaller exercise groups were formed with a limited number of attendees and plenty of space for each person. This structure differed from that used when the large groups had previously been arranged. At that time, large groups were perceived as the most favourable when arranging group exercise via the meeting places, however, this perspective changed when the smaller groups were formed. The smaller groups were more important than previously understood. They were valuable to the older persons who participated, and the small scale increased the possibility of an environment that invited conversation and personal contact with the older person. This made it easier to pay attention to all the older participating persons and to give everyone the time they needed. The smaller groups also provided an opportunity to talk with the older persons about their wishes and suggestions for improvement regarding the group exercise. These new insights brought another understanding that it was important not to cancel activities due to few participating older persons, which could have happened before the pandemic. Now the understanding was that it was important, for everyone who wanted to participate, that the exercise took place.

Ip2: And indeed, something good has come out of the pandemic; these smaller groups… it has become easier to see all the participants and everyone can get the time they really need.

M: Thanks to smaller groups?

Ip2: Smaller groups, yes.

M: Yes.

Ip5: I then thought spontaneously when you told me there were only four persons allowed to take part in a group at the beginning, it must still be nice for those who easily fall into the background of larger groups.

Ip2: Yes.

Ip5: And in this way get closer to each other and dare to offer more of themselves.

Ip2: Yes, it’s easier to become part of a context.

Ip5: Mm.

Ip2: And it’ll come too, if you talk about loneliness, involuntary loneliness. There are many people who aren’t comfortable being in large groups … and then, being someone in a small group makes them loosen up.

Ip1: Absolutely.

(Focus group no. 3)

### From deadlock to action

The stakeholders described a process which, for the older persons, initially represented a deadlock in terms of the ceased possibility to exercise via the meeting places, but which changed into an ability to act on the part of the older persons. This new situation caused the stakeholders concern about how it would affect the well-being of the older persons who used to exercise via the meeting places. In contrast, the stakeholders described how the older persons further into the pandemic showed an ability to act on their own initiative and took responsibility for the new situation. Such initiative brought happiness to the stakeholders.

#### Concern about the well-being of the older persons

The stakeholders described a concern that the lack of exercise via the meeting places would negatively affect the well-being of the older persons. The concern was based on what the older persons conveyed to the stakeholders in terms of inactivity and loneliness, which are factors the stakeholders were well aware could affect physical, mental as well as social well-being.

As for physical well-being, the older persons themselves conveyed to the stakeholders that they experienced aches, felt weaker and their fitness had deteriorated during the time without exercise via the meeting places. The stakeholders perceived that the older persons had become much older in a short period of time and that there was a difference between those who had only participated in sedentary activities at the meeting places before the pandemic compared to those who had developed good exercise habits via the meeting places before the pandemic.

Ip 6: What I hear from the coordinators [at the meeting places] is that they say… those who haven’t participated, just as you said, in the physical activities, who [instead] have participated in perhaps more sedentary activities, that many have weakened very quickly this year, you see how frail they have become.

Ip 7: Mm.

Ip 6: So, they haven’t felt good about being so isolated.

Ip 7: Mm.

(Focus group no. 1)

The mental well-being of the older persons was also perceived by the stakeholders to have been affected during the pandemic. Some older persons conveyed to the stakeholders that they had been isolated and had hardly met anyone during the entire pandemic. The isolation and not being able to be physically active via the meeting places was found to have made some older persons passive and inactive.

Ip 18: (…) So, we think that this Corona meant passivity for the older members and in some way, they can’t stand to get started again.

Ip 22: Mm.

Ip 18: So that …

M: Is this something the rest of you recognise?

Ip 22: Yes, so I agree that these older ladies who were there before the pandemic, basically almost none of them have come back, but there have been new, new participants. I agree with you, I think that they… they have in some way perhaps sunk into despair, I almost think.

Ip 18: I can imagine that some are also afraid of Corona infection after all, because it still flutters around a bit in the air, and there may be a risk with it. That’s the only reason we can think of.

Ip 22: Mm.

(Focus group no. 5)

The social well-being of the older persons was also perceived by the stakeholders to be affected. The social fellowship that accompanies the group exercises via the meeting places disappeared with the closing of the meeting places. The stakeholders described how the older persons communicated that they felt lonely and sad. When the group exercises were reopened to a certain extent, social interaction resumed, which was found to be of great value to those able to participate.

Furthermore, the stakeholders expressed concern that a general deterioration in the well-being of the older persons could become an obstacle for them to start exercising again via the meeting places, and they were also concerned about whether these needs could be taken care of at the meeting place. It was seen as a challenge to try to motivate them to come back to the group exercise again, as well as to support them in strengthening their well-being again. This was perceived as important work that needed to be initiated as soon as possible.

#### Happiness with the initiatives from the older persons

As the meeting places decreased or ceased to offer group exercises, the stakeholders said that some older persons continued exercising together in a group on their own initiative, which for the stakeholders brought happiness with the older persons’ ability to act. Such initiatives by the older persons themselves led to new opportunities for group exercise during the pandemic. The older persons arranged their own exercise sessions, which showed their strong will and desire to continue exercise in a group. Such initiatives were perceived to strengthen the independence of the older persons. Therefore, this was something that the stakeholders wanted to continue to encourage and support.

M: Mm, [Ip4], do you have any examples of whether you can notice a difference in their health?

Ip4: No more than that they themselves say they feel much better [when they exercise]. Now, I met someone the other day who said: “Ugh, it’s so boring, I can hardly stand on my feet” they said. “We have to get started”. “Yes”, I said, “but we can’t”. But they started the walking group on their own, so they walked by themselves. Even so, they started [the walking group] anyway in addition.

M: But you notice [a difference in their health] so you just see that. We don’t have any evaluation or anything. But do I understand you correctly? They have continued or started up walks themselves even though you cannot arrange it now during [the pandemic]?

Ip4: Yes, they gather here outside [the meeting place] every Monday and then they leave and then they come back after an hour, so I see them up and running, but I can’t come along.

Ip2: It’s great that they do it themselves.

[All agree]

Ip4: Yes, so there will be a group that can manage itself without me then.

Ip2: Yes, exactly.

(Focus group no. 3)

With the smaller exercise groups that were formed during the pandemic, came the need for more leaders to be engaged, which in turn led to the need to involve new volunteers. On the one hand, the stakeholders themselves recruited volunteers by asking them, and on the other hand, when older persons who themselves participated in a group exercise saw the need for more leaders, some volunteered to help with the exercise. This was perceived as very positive both by the stakeholders and by the individual volunteers who were happy to contribute, and thus allowed more opportunities to exercise. The positive response from the individual volunteers raised new ideas among the stakeholders about how to further support these new and powerful initiatives to optimize group exercise via the meeting places.

## Discussion

This study aimed to explore involved stakeholders’ experiences of group exercises for older persons arranged via municipal meeting places during the Covid-19 pandemic. The stakeholders experienced that the Covid-19 pandemic brought both opportunities and challenges in arranging the group exercises. Furthermore, the re-arranging of group exercises created concerns about the well-being of the older persons but also happiness with the older persons’ ability to act for their own well-being during the pandemic.

Inequality between municipalities in terms of access to health-promoting interventions seems to affect the well-being of older persons. The stakeholders perceived an openness in interpreting the guidelines from the authorities, resulting in municipalities making different decisions regarding the group exercises. This meant that depending on the municipality, the exercise either continued in adapted or new ways via the meeting places or was ceased. When it ceased, the stakeholders noticed negative effects on the physical, mental, and social well-being of the older persons. The difference regarding the exercise opportunities between the municipalities demonstrates inequality in health. The existence of inequality in health is well known globally (Folkhälsomyndigheten, 2020b; WHO, 2008). According to Scharf et al. (2017) inequality in later life is significantly influenced by where the older person lives, their health and income. But when efforts were made to counteract inequality in later life, good effects were seen (Scharf et al., 2017). For example, for those having a visual and hearing impairment, Henni et al. (2022) demonstrated the importance of involving them at an early stage when designing digital health interventions to reduce inequality (Henni et al., 2022). However, to succeed requires an increased focus on inequalities at the local level, and the importance of finding new solutions and learning from each other through exchanging information on what works in other places is emphasized in order to reduce inequalities (Folkhälsomyn digheten, 2020b; Scharf et al., 2017). The stakeholders in the present study emphasized the importance of making adapted and local decisions. Accordingly, an exchange of experiences between the municipalities could have reduced the risk of interpreting the guidelines from the authorities in different ways and could have reduced the inequality related to group exercise between the municipalities. In addition, Petriwskyj et al. (2012) highlight the importance of letting the older persons participate in decision-making on a local governance level. It is important that the local governance, the level of authority closest to the people, enables older persons to be included and becomes involved in decisions at the local level (Petriwskyj et al., 2012). Such societal participation on a group level is an essential component of active ageing (WHO, 2002), health promotion (Naidoo & Wills, 2016) and a person-centred approach (McCormack & McCance, 2017). In the present study an indirect participation was revealed, where the decision to reopen the meeting places was partly based on the experiences and perceptions of the participating older persons and what they had conveyed to the stakeholders. However, during the pandemic, collaboration between the stakeholders and the older persons in decision-making at a municipal level could have reduced the older persons’ perceived negative impact on health and decreased inequalities between municipalities.

Despite aggravating circumstances, adaptations and new solutions can enable development and improvement. The smaller groups arranged in this study were perceived as a quality improvement. The small size implied a more person-centred encounter with an environment that invited conversation and personal contact with the older persons; everyone was seen and listened to. This is in line with person-centredness which is an approach based on relationships where the person is at the centre (McCormack & McCance, 2017). Nivestam et al. (2021) showed the importance of a person-centred approach by recognizing the person. This was achieved by the older persons feeling of being listened to, which also led to the experience of empowerment (Nivestam et al., 2021). The person-centred encounter that the smaller groups in this study entailed provided an opportunity to get first-hand information from the older persons about their wishes and suggestions for improvement regarding the group exercise, which increased the older persons’ ability to be involved. The importance of involving local older persons to design, develop, implement and promote important physical activities in their context is highlighted by Boulton et al. (2020). This study’s findings show that a person-centred encounter enables the involvement of the older persons regarding the group exercise via the meeting places. An increased collaboration with the older persons will improve the prerequisites for developing and improving physical activities via the meeting places. However, more studies are needed that explore how to further enable a person-centred encounter with the older persons when exercising in a group and whether this could lead to more customized activities according to the wishes of the older persons.

During a challenging period, the capacity, resources and drive of older persons were made visible. The stakeholders said that older participating persons voluntarily offered to lead group exercise when there was a lack of leaders. This initiative was perceived as very positive, both by the stakeholders and by the individual volunteers who were happy to contribute and thus enabled more occasions to exercise. The positive effects of volunteering are highlighted by Anderson et al. (Anderson et al., 2014), who showed that psychosocial, physical and cognitive health, as well as functional performance, improved in older persons when volunteering. Furthermore, Gonzales et al. (Gonzales et al., 2015) showed that by recruiting and retaining older volunteers, certain costs associated with volunteering, such as the cost of education and support and personal costs such as lost time for work, decrease (Gonzales et al., 2015). Seeing the older person as a valuable resource is highlighted in the present study through the stakeholders describing this ability to act as something positive, and the fact that volunteering was something that the stakeholders wanted to continue to support after the pandemic. When the meeting places ceased to offer group exercise, some older persons themselves took the initiative to continue exercising together. Hartley and Yeowell (Hartley & Yeowell, 2015) described how some older persons took on an organizational role in the group exercise as a way of participating. They proposed that older persons should be given the right of ownership, so they could lead their own exercise groups. This would be beneficial as it could lead to the needs of the group being more easily met, but it could also be empowering for the older person when having responsibility for the group (Hartley & Yeowell, 2015). Having the starting point that each person has the power, resources and capacity to self-identify their own needs and to find strategies to manage these, i.e., empowerment, is an important part of health promotion (WHO, 1986) and person-centredness (McCormack & McCance, 2017). This study brings an understanding of the importance of stakeholders, via the meeting places, strengthening supportive environments by sharing the ownership of arranging the group exercises with the older participating persons.

### Methodological considerations

The trustworthiness of this study will be discussed by using the four concepts of credibility, dependability, confirmability and transferability according to Lincoln and Guba (Lincoln & Guba, 1985).

To achieve credibility, i.e., to ensure the findings were consistent with reality, a varied selection of stakeholders who had experience in arranging group exercise via the meeting places was sought (Lincoln & Guba, 1985; Shenton, 2004). For a varied sample, each participating municipality was contacted to find everyone who was involved in the group exercise via the meeting places, which resulted in a selection of 25 stakeholders with varying backgrounds. However, credibility could have been further strengthened by increasing the breadth of interviewed stakeholders, selecting from additional municipalities for an even more varied sample. Furthermore, to explore the stakeholders’ experiences it was important that they were able to express themselves freely during the focus group interview. This was achieved partly by choosing homogeneous focus groups, as a common frame can facilitate the interaction, and partly by offering online support regarding the use of video conference systems before the interview. Such support was considered a strength as no one was excluded because of insufficient technical knowledge. Additionally, the online focus groups were considered a strength as it was easier to participate for practical reasons and this thereby facilitated recruitment. Furthermore, credibility can be affected if the focus groups either have too many or too few participants. The focus groups had three to five stakeholders. Krueger and Casey (Krueger & Casey, 2015) recommend groups of four to eight participants, but this applies to focus groups where the participants meet physically. For online focus groups, it can be an advantage if they are smaller as small group sizes can be easier to manage (Daniels et al., 2019; Kite & Phongsavan, 2017). However, the dialogue and interaction in all focus groups worked well, since all stakeholders were involved and shared their experiences. To verify the findings a member check with the stakeholders was conducted. The member check included an oral presentation and a discussion of the preliminary findings, which the stakeholders confirmed. The stakeholders had the possibility to add anything they perceived to be missing in the findings, but nothing new was raised (Lincoln & Guba, 1985).

To increase the study’s dependability, i.e., to ensure the findings are consistent and can be repeated, the context, description of the stakeholders, data collection and analysis have been clearly presented. This will enable the study to be repeated in a similar context and with similar participants and to produce similar findings (Lincoln & Guba, 1985; Shenton, 2004).

To achieve confirmability, i.e., ensuring neutrality by preventing the findings from being biased by the researchers’ perspectives, the first and last authors, due to their acquaintance with two stakeholders, chose not to participate in two of the focus groups. Also, the analysis was conducted by several researchers and the findings were discussed and continuously revised to ensure that they reflected the stakeholders’ experiences and not the researchers’ perspective (Lincoln & Guba, 1985; Shenton, 2004).

To achieve transferability, i.e., the possibility that the findings can be transferred to another context and other groups, this study’s context and stakeholders have been thoroughly described (Lincoln & Guba, 1985; Shenton, 2004). This study explored experiences during a global pandemic, which narrows transferability after the pandemic. However, the study’s results may be used in the event of a similar global event or locally if a municipality’s meeting place can no longer offer group exercise for older persons. Additionally, the findings can be used to further develop the health-promoting interventions arranged via the meeting place.

## Conclusion

In conclusion, this study showed opportunities and challenges when arranging group exercise for older persons during the pandemic. The findings of this study highlighted the importance of reducing inequalities in health in new situations such as a pandemic, exchanging experiences between the municipalities, and making the older persons more involved in the decision-making process. During the pandemic, involving the older persons could have been a way to reduce the perceived negative impact on the older persons well-being.

This study demonstrated the older persons’ ability to act and highlighted the importance of strengthening supportive environments by sharing the ownership of arranging the group exercises with the older participating persons.

Also, this study has contributed understanding of the importance of a person-centred encounter with the older persons when exercising in groups, as this increased the opportunities for the older persons to be seen and to be involved. However, more studies are needed that explore how to further enable a person-centred encounter with older persons when exercising in groups.

## Supplementary Material

Supplemental Material

## Data Availability

The data that support the findings of this study are available from the corresponding author, [D.D.], upon reasonable request.
