# Supplementary material for: The influence of the Covid-19 pandemic on municipal meeting places arranging group exercise for older persons
Source: Int J Qual Stud Health Well-being. 2023 Jul 27;18(1):2235130. doi: 10.1080/17482631.2023.2235130 (PMC10375932; doi:10.1080/17482631.2023.2235130)
Supplement: Supplemental Material [file ZQHW_A_2235130_SM6048.docx]

**Supplementary file 1: Interview guide**

## Introduction

All participants are asked to briefly present themselves and their role in arranging group exercise via the meeting places.

## Opening interview request

- Please tell us about the group exercise activities that you are involved in via the municipal meeting places.

## Challenges during the pandemic

- Were your group exercise activities affected during the pandemic? If so, how?

- How did you as a stakeholder handle the transition that the pandemic required?

## Opportunities and lessons learned during the pandemic

- What response have you received to the group exercise activities from the older persons during the Covid-19 pandemic? What do they wish for in this new situation?

- Have lessons been learned from the pandemic that you will use when designing new group exercise activities? Or when modifying existing group exercise activities?

- If there were to be a new pandemic, how would you as a stakeholder act?

## Closing interview question

- Is there anything important that you think we have omitted to discuss?

## Examples of prompting questions:

Can you give an example…?

What do you mean when you say…?

Can you tell me in which way…?
